# Analysis of LINE-1 DNA Methylation in Colorectal Cancer, Precancerous Lesions, and Adjacent Normal Mucosa

**DOI:** 10.3390/medicina61071243

**Published:** 2025-07-10

**Authors:** Inga Kildusiene, Ryte Rynkeviciene, Auguste Kaceniene, Rima Miknaite, Kestutis Suziedelis, Giedre Smailyte

**Affiliations:** 1 Institute of Biosciences, Life Sciences Center, Vilnius University, LT-03101 Vilnius, Lithuaniakestutis.suziedelis@gf.vu.lt (K.S.); 2 Laboratory of Cancer Epidemiology, National Cancer Institute, P. Baublio 3B, LT-08406 Vilnius, Lithuania; auguste.kaceniene@nvi.lt (A.K.); giedre.smailyte@nvi.lt (G.S.); 3 Laboratory of Molecular Oncology, National Cancer Institute, Santariskiu 1, LT-08406 Vilnius, Lithuania; ryte.rynkeviciene@nvi.lt; 4 Institute of Health Sciences, Faculty of Medicine, Vilnius University, LT-03101 Vilnius, Lithuania

**Keywords:** *LINE-1*, colorectal cancer, methylation

## Abstract

*Background and Objectives*: Colorectal cancer (CRC) is a major cause of cancer morbidity and mortality worldwide. Genetic and epigenetic changes, especially DNA methylation alterations, are key in CRC development. *LINE-1* hypomethylation marks global DNA methylation loss and genomic instability, making it a potential early CRC biomarker. This study investigates the methylation status of *LINE-1* in colorectal adenocarcinoma, precancerous lesions (tubular and serrated adenomas), and the surrounding normal mucosa, aiming to elucidate its role as an epigenetic marker in early colorectal tumorigenesis. *Materials and Methods*: Paired lesion and normal tissue samples from 66 patients were analyzed for LINE-1 methylation at three CpG sites using bisulfite pyrosequencing. *Results:* Adenocarcinomas and tubular adenomas showed significant hypomethylation, especially at loci A and B, while serrated adenomas exhibited no significant differences. *Conclusions*: *LINE-1* hypomethylation is associated with colorectal tumorigenesis, with distinct patterns observed between tubular and serrated adenomas, indicating distinct pathways forming and progressing specific adenomas. These findings support the potential of *LINE-1* methylation as an early epigenetic biomarker for CRC risk stratification and highlight the need for further research into its clinical utility.

## 1. Introduction

Colorectal cancer (CRC) is the third most common cancer worldwide, with over 1.15 million new cases and 576,000 deaths annually [1]. CRC develops through a multistep process driven by genetic and epigenetic alterations, primarily via three pathways: chromosomal instability (CIN, ~85%), microsatellite instability (MSI, ~15%), and CpG island methylator phenotype (CIMP, ~25%) [2,3].

Epigenetic mechanisms—DNA methylation, histone modifications, and non-coding RNAs—regulate gene expression without altering the DNA sequence [4,5,6,7]. DNA methylation, especially at CpG islands in gene promoters, is the most studied in CRC and is linked to gene silencing, carcinogenesis, and potential biomarker development [8].

Histone modifications and non-coding RNAs also influence gene regulation and cancer progression [9,10,11]. Notably, DNA methylation changes, including hypermethylation of tumor suppressor genes and global hypomethylation, often occur early in CRC, even in adjacent normal mucosa, reflecting an epigenetic “field effect” [12,13,14,15,16,17,18].

*LINE-1* retrotransposons are normally silenced by methylation to maintain genomic stability. Their hypomethylation is common in CRC and precancerous lesions, correlating with genomic instability and cancer risk. *LINE-1* methylation thus serves as a marker for global methylation status and early CRC detection [19,20].

This study examines *LINE-1* methylation in colorectal cancer, precancerous lesions (tubular and serrated adenomas), and adjacent normal tissue to assess its potential as an early biomarker for CRC.

## 2. Materials and Methods

### 2.1. Study Population

From January to April 2021, 66 patients undergoing a colonoscopy screening at our institution were prospectively enrolled. Inclusion criteria were age 50–74 years, positive fecal immunochemical test (FIT), absence of gastrointestinal symptoms, and no active infections.

### 2.2. Tissue Collection and Classification

Paired samples were taken during colonoscopy from lesions (tubular adenoma >10 mm, serrated adenoma, or adenocarcinoma) and adjacent normal mucosa. Lesions were classified histologically. The study included 30 tubular adenoma, 15 serrated adenoma, and 21 colorectal adenocarcinoma patients.

### 2.3. LINE-1 Methylation Analysis

All samples were stored in RNAlater (Qiagen, Hilden, Germany) at −80 °C. DNA was extracted with QIAamp Mini Kit (Qiagen, Hilden, Germany) and quantified by Nanodrop 2000 (Wilmington, DE, USA). Bisulfite conversion used 500 ng DNA and the EpiTect Kit (Qiagen, Hilden, Germany). LINE-1 methylation was analyzed by pyrosequencing (PyroMark Q24 LINE-1 kit, Qiagen, Hilden, Germany) targeting three CpG sites.

Bisulfite DNA was PCR-amplified using a PyroMark PCR Kit (Qiagen, Hilden, Germany) with 12.5 µL Master Mix, 2.5 µL CoralLoad, 0.5 µL primers, 2 µL DNA, and 7 µL water. PCR: 95 °C 15 min; 45 cycles (94 °C 30s, 50 °C 30 s, 72 °C 30 s); final 72 °C 10 min. Products were checked on 1% agarose gel.

For pyrosequencing, 20 µL biotin-labeled PCR product was processed with Sepharose beads, the primer was added, and the product was then analyzed on PyroMark™ Q96 system (Qiagen, Hilden, Germany).

### 2.4. Statistical Analysis

Methylation levels at three *LINE-1* CpG loci (A, B, C) were quantified and expressed as mean ± standard deviation (SD) values. Paired *t*-tests compared methylation between pathological and adjacent normal tissues within each lesion type. An ROC analysis was performed using Graphpad Prism 8. A *p*-value < 0.05 was considered statistically significant.

## 3. Results

We performed a *LINE-1* DNA status analysis in three promoter CpG loci (A, B, C) in pathological tissue (tubular adenoma (TA), serrated adenoma (SA), or adenocarcinoma) and surrounding tissue. These loci correspond to cytosine positions 328, 321, and 318 within the CpG islands of the gene X58075.1.

**Figure 1:**
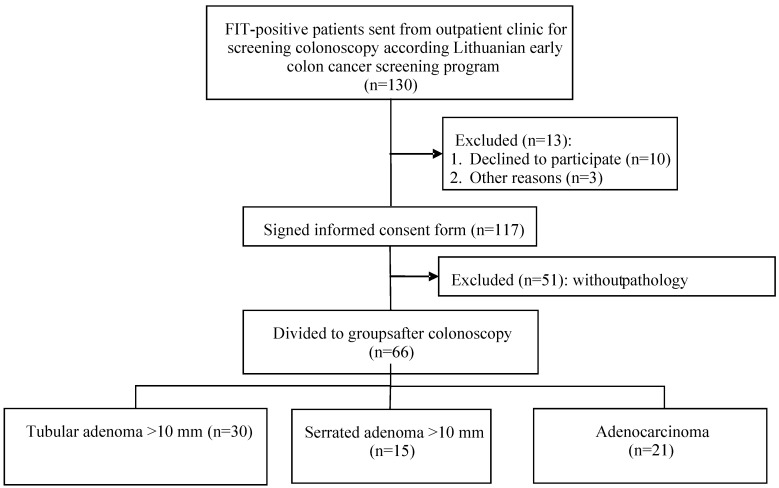
Study patients.

This study revealed statistically significant differences in methylation levels between different CpG islands. In this study 66 FIT positive patients were selected (Figure 1): 30 samples from TA (36.3%), 15 from SA (23.9%) and 21 from adenocarcinoma (29.9%). The mean age of patients was 62.9 ± 6.3 for TA, 59.9 ± 5.0 for SA, and 65.7 ± 7.1 for adenocarcinoma. Demographic variables were selected to ensure approximate equivalence across groups.

### 3.1. LINE-1 Methylation in Pathological and Adjacent Tissues

**Locus A**

- TA: *LINE-1* methylation was lower in adenoma (72.53 ± 1.48) than normal mucosa (81.63 ± 0.71; *p* < 0.05).
- SA: No significant difference between lesion (78.67 ± 1.11) and normal tissue (79.6 ± 0.58).
- Adenocarcinoma: Tumor tissue showed reduced methylation (66.33 ± 2.24) vs. mucosa (83.76 ± 0.76; *p* < 0.05).

**Locus B**

- TA: Lower methylation in lesion (67.53 ± 1.26) than normal tissue (70.97 ± 0.91; *p* < 0.05).
- SA: No significant difference.
- Adenocarcinoma: Tumor tissue (64.43 ± 2.61) showed significantly less methylation than normal mucosa (73.24 ± 0.72; *p* < 0.05).

**Locus C**

- TA: Lesion methylation (67.07 ± 1.53) was lower than normal tissue (72.27 ± 1.15; *p* < 0.05).
- SA: No significant difference.
- Adenocarcinoma: Tumor tissue (65.05 ± 1.70) had significantly reduced methylation compared to normal mucosa (72.76 ± 0.90; *p* < 0.05).

### 3.2. Comparative Analysis

**Figure 2:**
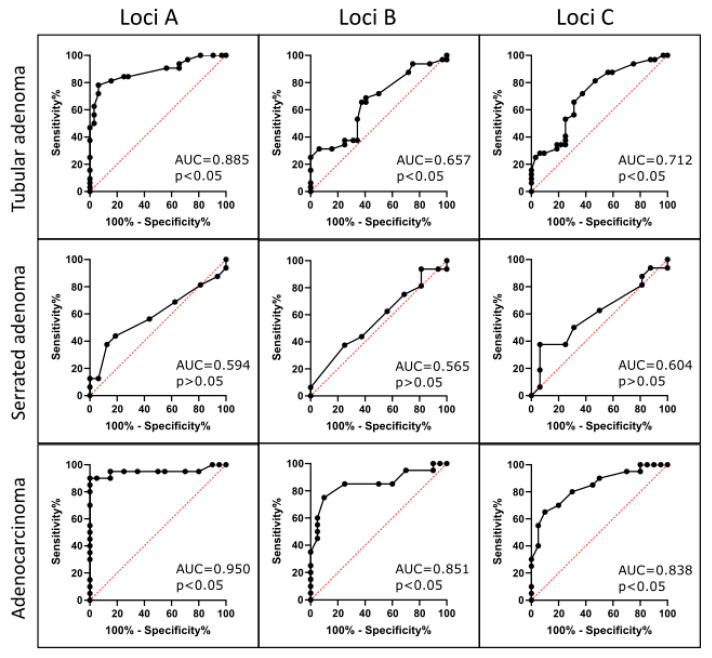
ROC curve analysis of LINE-1 methylation at three CpG sites for discriminating tubular adenoma, serrated adenoma, and adenocarcinoma from adjacent healthy tissue. Red dotted line indicates random classifier. Black line indicates ROC curve.

- The ROC analysis revealed statistically significant differences in methylation levels among the three CpG loci (Figure 2 and Table 1).
- Adenocarcinoma tissues consistently exhibited the lowest *LINE-1* methylation across all loci, with the greatest difference at locus B.
- Tubular adenomas showed intermediate hypomethylation, with the most pronounced difference at locus A.
- Serrated adenomas did not display significant methylation differences between pathological and normal tissues at any locus.

### 3.3. Summary of Findings

- *LINE-1* hypomethylation is most pronounced in adenocarcinoma, less so in tubular adenoma, and minimally in serrated adenoma.
- The largest methylation differences were observed at locus A, suggesting its potential as a focal point for future biomarker studies (Table 2).
- No significant methylation changes were detected in serrated adenomas, indicating possible differences in their tumorigenic pathways compared to tubular adenomas and adenocarcinomas (Table 2).

## 4. Discussion

### 4.1. The Role of DNA Methylation and LINE-1 in Colorectal Carcinogenesis

CRC develops through genetic mutations and epigenetic changes, especially DNA methylation. Normally, *LINE-1* is heavily methylated to prevent harmful activity, but in tumors, hypomethylation reactivates *LINE-1*, causing mutations and instability [21,22]. This study examined methylation at three *LINE-1* promoter CpG sites using a commercial kit, focusing on overall methylation and the importance of specific loci.

### 4.2. Distinct Pathways in Serrated Versus Tubular Adenomas

Our study found SAs lack significant *LINE-1* hypomethylation versus normal tissue, unlike TAs and carcinomas, indicating distinct pathways. SAs involve *BRAF* mutations, CIMP, and MSI; tubular adenomas show chromosomal instability and global hypomethylation [23]. This limits *LINE-1* methylation’s role in SA risk assessment. Our findings stress the need to study gene-specific methylation for better CRC biomarkers. Since SAs may progress faster, current screening intervals based on TAs might need revision. Detailed methylation research and blood-based markers could improve screening and enable non-invasive detection.

### 4.3. Limitations and Future Directions

This study offers valuable insights but is limited by the small sample size, focus on FIT-positive patients, and lack of a healthy control group, making it hard to distinguish cancer-specific methylation changes from normal variation. Including healthy controls was unfeasible due to the invasive nature of colonoscopy. Future research should use larger, diverse cohorts with a longitudinal follow-up, integrate molecular and clinical data, and include functional studies to clarify *LINE-1* hypomethylation’s role in tumorigenesis and its reversibility.

## 5. Conclusions

In summary, our results reinforce the central role of *LINE-1* hypomethylation in the pathogenesis of CRC, particularly within the conventional adenoma–carcinoma sequence. The locus-specific analysis highlights the potential of targeted methylation assays for early detection and risk assessment. The distinct epigenetic profiles of serrated and tubular adenomas underscore the need for subtype-specific biomarkers and personalized approaches to CRC prevention and management.

## Figures and Tables

**Table 1:** ROC analysis summary for CpG loci methylation levels. ROC, receiver operating characteristic; AUC, area under receiver operating characteristic curve; CI, confidence interval.

	AUC	Std. Error	95% CI		*p* Value
TA (Loci)					
A	0.885	0.043	0.80	0.97	<0.0001
B	0.657	0.069	0.52	0.79	<0.05
C	0.712	0.065	0.59	0.84	<0.01
SA (Loci)					
A	0.594	0.104	0.39	0.80	>0.05
B	0.565	0.103	0.36	0.77	>0.05
C	0.604	0.102	0.40	0.80	>0.05
Adenocarcinoma (Loci)					
A	0.950	0.043	0.87	1.00	<0.0001
B	0.851	0.065	0.72	0.98	=0.0001
C	0.838	0.063	0.71	0.96	<0.001

**Table 2:** *LINE-1* methylation levels in CpG island A.

	Number	Mean ± SD	95% CI	
Adenocarcinoma	21			
Pathological		66.33 ± 10.27	61.66	71.01
Surrounding		83.76 ± 3.49	82.17	85.35
Tubular adenoma	30			
Pathological		72.93 ± 7.73	70.04	75.82
Surrounding		81.77 ± 4.03	80.26	83.27
Serrated adenoma	15			
Pathological		78.67 ± 4.03	76.28	81.05
Surrounding		79.6 ± 2.23	78.37	80.83

## Data Availability

The datasets used and/or analyzed during the current study are available from the corresponding author on reasonable request.
